# Biomechanical evaluation of two minimal access interbody cage designs in a cadaveric model

**DOI:** 10.1186/s40634-018-0165-1

**Published:** 2018-12-19

**Authors:** D. Kok, C. M. M. Peeters, F. H. Wapstra, S. K. Bulstra, A. G. Veldhuizen

**Affiliations:** 1 Department of Orthopaedics, Rijnstate Hospital, Wagnerlaan 55, 6815 AD Arnhem, The Netherlands; 2 Department of Orthopaedics, University of Groningen, University Medical Center Groningen, Groningen, The Netherlands; 3 Department of Orthopaedics, Rijnstate Hospital, P.O. Box 9555, 6800 TA Arnhem, The Netherlands

**Keywords:** Interbody fusion, Cages, Spinal fusion, Memory metal minimal access cage, Nitinol

## Abstract

**Background:**

Different interbody grafts have been employed and evaluated for spinal fusion surgery. The Memory Metal Minimal Access Cage (MAC) is a hollow horseshoe shaped interbody fusion concept which provides a potentially major advantage with their small cage contact area and large graft space in comparison with other vertical cages.

**Methods:**

This Biomechanical Cadaveric Study evaluates the primary stability and the amount of acute subsidence occurring in two new MAC cage designs; the Niti-l and Niti-s. Both cages were made of nitinol in the form of a wedge-shaped horseshoe with spikes on the edges. Differences were the higher weight and larger tranverse section area of the Niti-l due to his specific design with two different layers of thickness. Biomechanical axial compression tests were performed on ten fresh-frozen T11-L5 vertebral bodies.

**Results:**

A direct relation between force at failure and BMD was found (*p* < 0.001). The displacements in the vertebral body at an axial force of 800 N were 1.91 mm and 1.88 mm for the NiTi-l and NiTi-s cage, respectively. The mean failure load for the NiTi-l cages was 2043 N, and 1866 N for de NiTi-s cages. No significant difference was established between the two cages.

**Conclusion:**

The biomechanical strength of both NiTi-l and NiTi-s cages is good and comparable to each other with a limited amount of short-term subsidence after the initial implantation of the cage spikes into the bone.

## Background

Posterior lumbar interbody fusion (PLIF) is introduced independently in the 1940s by Jaslow and Cloward for the treatment of refractory discogenic back pain (Cloward, 1953; Cloward, 1963; Cloward, 1981; Jaslow, 1946). The surgical goals of PLIF are to immobilize the unstable degenerated intervertebral disc area with direct neural decompression, to restore normal disc height, to provide segmental alignment and balance, and to restore load-bearing to anterior structures (Panjabi, 1988; Wang et al., 2014).

**Fig. 1:**
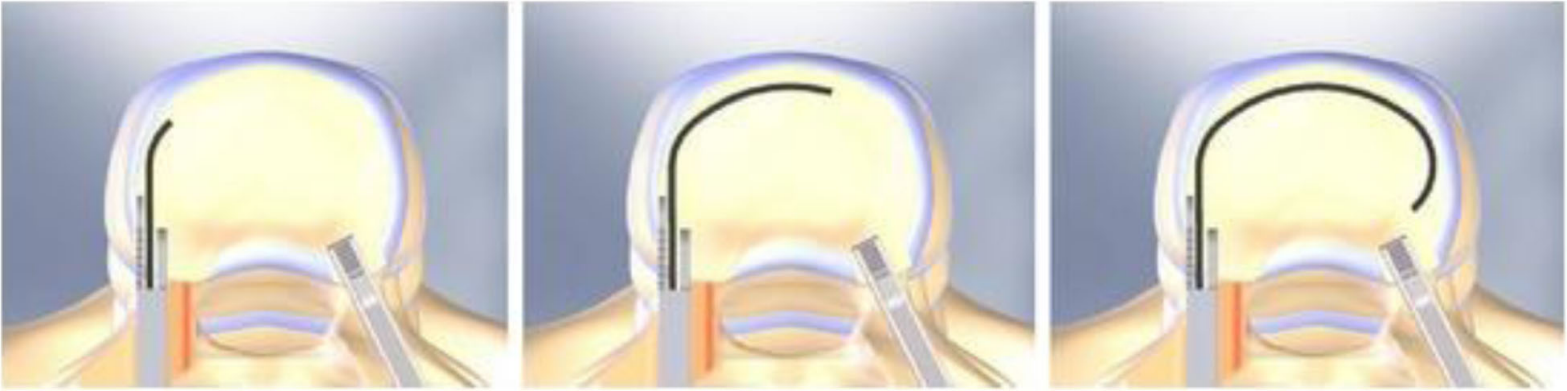
Implantation technique MAC cages

Different interbody grafts have been employed and evaluated in the last decades. Generally good results have been reported for allogeneic or autogenous corticocancellous interbody bone grafts (Loguidice et al., 1988). However, the use of these bone grafts alone leads to several limitations in biomechanical strength and donor-site morbidity (Fernyhough et al., 1992; Loguidice et al., 1988; Pfeiffer et al., 1996; Soini, 1994; Younger & Chapman, 1989). Interbody fusion cages, on the contrary, are thought to fulfil both mechanical and biological requirements for fusion as they are designed to withstand high axial loads without graft subsidence, and to allow insertion of bone graft or other osteoconductive materials (Boucher, 1959; Evans, 1985; Panjabi, 1988). Although the popularity of metal cages has increased rapidly, the mismatch in the elastic modulus between the cage and the vertebral bone can lead to stress shielding, resulting in a delayed fusion and increased risk of cage failure (Cunningham et al., 1997). Carbon fiber cages are closest to the elastic modulus of the vertebral body, but synovitis related to the carbon fiber debris has been reported (Parsons et al., 1985). Titanium implants, developed by Bagby and Kuslich, Ray, and Harms, also exhibit the necessary biomechanical strength and offer a radiopaque alternative to carbon fibre materials (Kuslich et al., 1998; Ray, 1997). Their open design provides large graft surface areas that allow for sufficient bone ingrowth.The Memory Metal Minimal Access Cage (MAC) builds on the developments made in PLIF procedures, but uses the experience gained from titanium mesh technology. The MAC cage is a hollow horseshoe shaped interbody fusion concept which can be inserted through a more minimal approach with the use of a new delivery system (DePuy Spine International). During implantation into the disc space the device deploys from a straight configuration into a curved configuration due to the shape memory of nitinol (Fig. 1). With this technique, the cage can be positioned well in the front, close to the cortex of the vertebral body. Once in-situ, the MAC cage has been thought to provide appropriate structural support to the concerned vertebral bodies. Another major advantage of this device is the relatively small cage contact area with the vertebrae. This results in larger spaces for additional bone grafts, what theoretically should lead to higher rates of solid fusion. Great biomechanical strength and high primary stability of the intervertebral device are important prerequisites for the translational application of new interbody cage designs in patients.Due to the relatively small cage contact area, this new device might not withstand the required axial load between two vertebral bodies of the human spine. Therefore, the aims of this biomechanical study were to evaluate the primary stability and the amount of acute subsidence occurring in two new MAC cage designs; the NiTi-l and NiTi-s. Our hypothesis was that the two MAC cage designs are comparable to each other.

## Methods

### Specimen preparation

Ten fresh-frozen T11-L5 vertebral bodies were obtained from the department of Pathology, for use in biomechanical testing of the two new MAC cage designs (Table 1). The intervertebral discs and remaining cartilaginous materials were removed down to the bony endplate. The use of human vertebral bodies was granted by the ethics committee. Dual-energy X-ray absorptiometry was performed to measure bone mineral density (BMD) of each vertebral body in order to obtain comparable specimens. Vertebrae were harvested within 3 days post mortem and immediately deep-frozen at − 20 °C.

**Table 1:** Characteristics of the specimens

Cage number	Spine number	Vertebra	Spongious BMD (g/cm^2^)	Cortical BMD (g/cm^2^)
1. NiTi-I	4	L4	125	367
2. NiTi-I	8	L3	75	300
3. NiTi-I	10	L4	75	323
4. NiTi-I	13	T11	133	231
5. NiTi-I	13	L5	133	231
Mean			108	290
SD			31	59
6. NiTi-s	4	L5	125	367
7. NiTi-s	8	T11	75	300
8. NiTi-s	8	L4	75	300
9. NiTi-s	13	L4	133	231
10. NiTi-s	17	L1	160	419
Mean			114	323
SD			38	72

### Devices

**Fig. 2:**
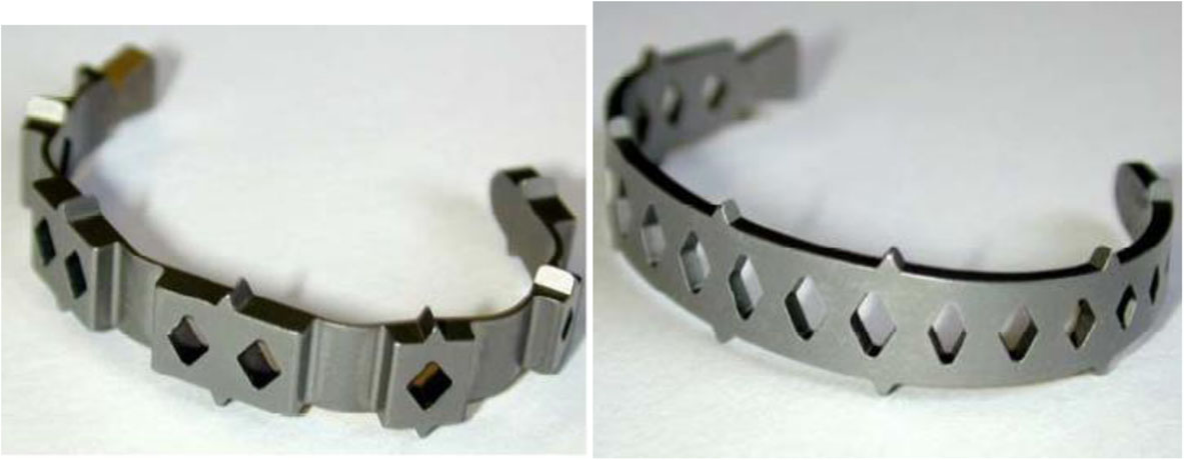
The new prototype cages (left: NiTi-l, right: NiTi-s)

In this study two MAC designs were tested: the NiTi-l and NiTi-s (Fig. 2). Both cages are made of nitinol in the form of a horseshoe and can be implanted from a posterior approach with the use of a new delivery system (DePuy Spine International), as shown in Fig. 1. Diamond shaped holes in the cage and a large graft surface area allow for good bone ingrowth between the affected vertebrae. The spikes on the edges and the wedge shape of the design provide optimal stability and device fitting. The thickness of the cages varies between 1.08 mm and 1.25 mm, depending on the size. Only small size devices with an overall height of 7 mm were tested in this study, including (i) two NiTi-l cages with a weight of 4.581 g and 4.580 g, a transverse section area of 30.4mm^2^, and a spike height of 1.2 mm, and (ii) two NiTi-s cages with a weight of 3.094 g and 3.105 g, a transverse section area of 15.04mm^2^, and a spike height of 1.1 mm. Besides differences in weight and transverse section area, a difference in the design exists between the NiTi-l and NiTi-s cages. The thickness of the NiTi-s cage is constant over the whole implant, whereas the NiTi-l cage has two different layers of thickness in their implant (Fig. 2). From this reason the NiTi-l cage has a larger transverse section area. Before biomechanical testing the devices were placed in a water bath of 60 °C to make sure that the material was in its super elastic phase.

### Axial compression tests

The biomechanical tests were performed with the use of a servo-hydraulic material testing system (MTS Bionix 858.2). The load was applied in force control mode with a constant loading rate of 50 N/s.

**Fig. 3:**
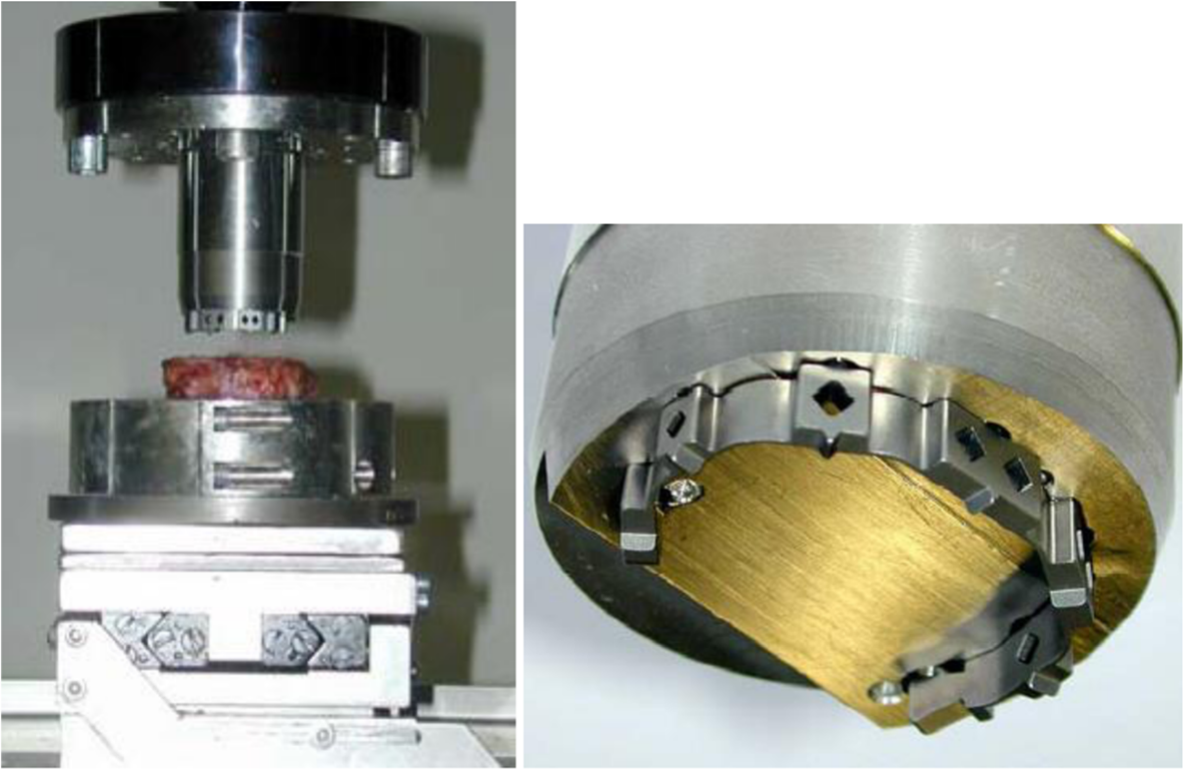
Setup for the axial compression test of the MAC cage designs

Compressive force and displacement data were electronically recorded. The actuator of the MTS was equipped with a metal cylinder which provided perfect fitting with the MAC cages in order to prevent stability failures during the load application (Fig. 3). Hot glue was used at three points for the attachment of the cage to the cylinder. The vertebral bodies were embedded in special holders using Polymethyl methacrylate (Technovit®) and placed on a XY-table. The cages were positioned approximately 3 mm behind the front of the vertebral bodies according to instructions of DePuy International. In some cases the vertebral endplate did not make contact with the entire interface prior to the test due to irregularity of the endplate. Subsidence was assessed at a load of 800 N. The compression test was continued until a subsidence of 7 mm occurred. The failure load was defined as the maximum load reached before fracture of the vertebral endplate.

### Statistical analysis

SPSS 9.3 software was used for the analysis of data, and statistically significant values were defined as *P* < 0.05. Comparison of subsidence at a load of 800 N and failure load was performed using analysis of covariance, taking BMD as covariance.

## Results

**Fig. 4:**
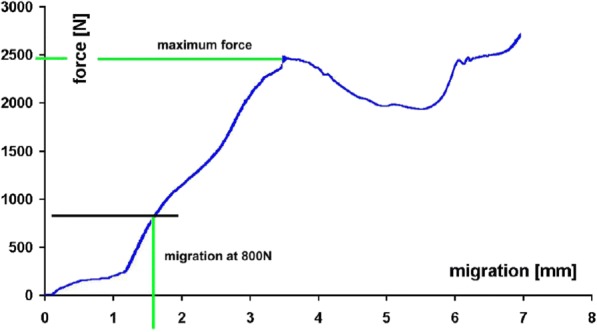
Typical axial load-displacement curve of a MAC cage

A compressive load-displacement curve for one MAC cage is shown in Fig. 4. A direct relation between force at failure and BMD was found (*p* < 0.001). At the beginning of the curve relatively little axial force was needed for 1,2 mm displacement in the vertebral body. This was explained by the implantation of the cage spikes into the bone, since their height were also about 1.2 mm. Subsequently, more axial force was needed for further displacement until the moment of fracture of the vertebral endplate.

**Fig. 5:**
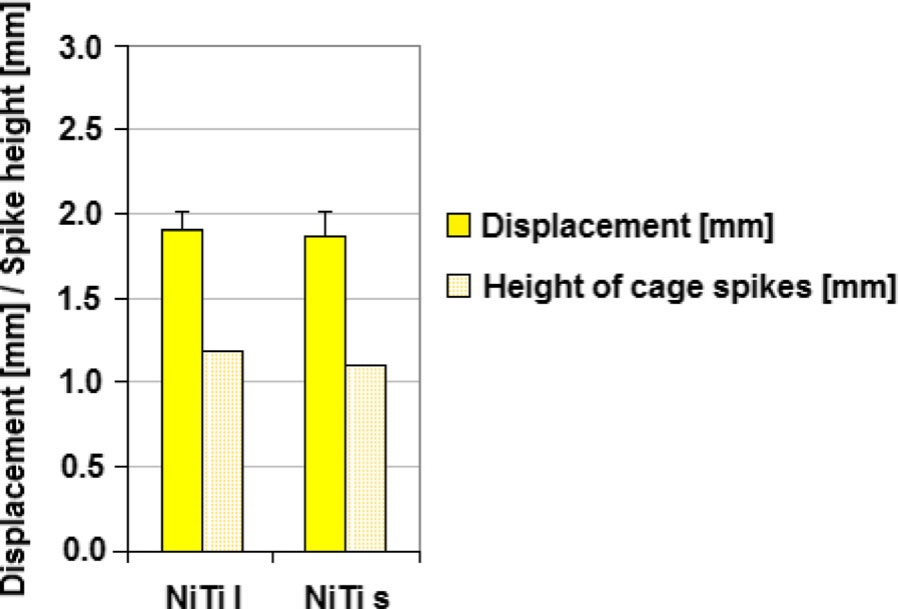
Mean displacement at a force of 800 N and spike height of the cage

The displacements in the vertebral body at an axial force of 800 N were 1.91 mm and 1.88 mm for the NiTi-l and NiTi-s cage, respectively. As Fig. 5 indicates, no significant difference was established between the two cages.

**Fig. 6:**
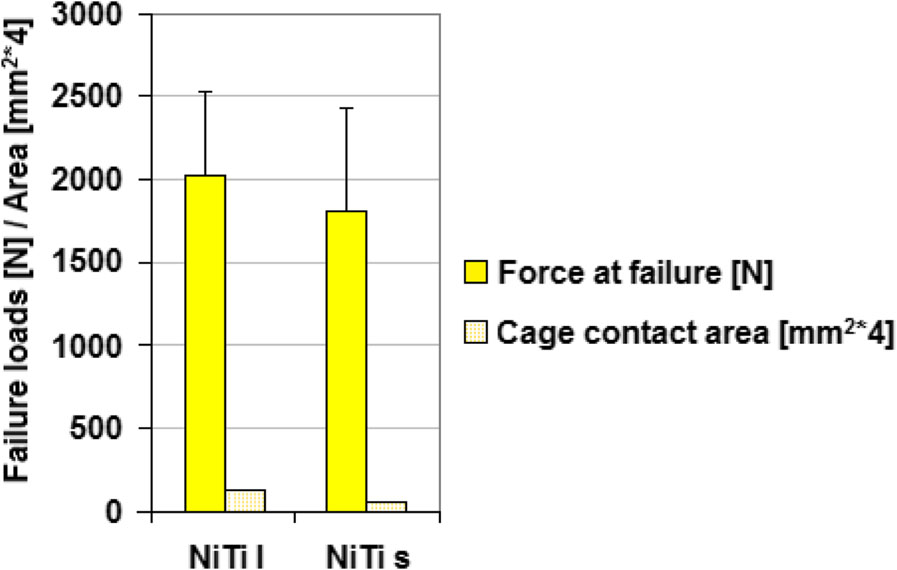
Maximum force together with cage contact area (means and standard deviation)

The failure load and cage contact area of both cages are presented in Fig. 6. The mean failure load for the NiTi-l cage was 2043 N, and 1866 N for the NiTi-s cage. The failure load of NiTi-l group was slightly higher than the NiTi-s group, but no significant difference was established.

## Discussion

The aims of this biomechanical study were to evaluate the primary stability and the amount of acute subsidence occurring in two MAC cage designs. A servo-hydraulic material testing system was used to perform the axial compression test.

The Niti-l and Niti-s were tested in this study. Both cages are made of nitinol in the form of a wedge-shaped horseshoe with spikes on the edges. Differences are the higher weight and larger tranverse section area of the Niti-l due to his specific design with two different layers of thickness. The acute subsidence at a force of 800 N was similar between the two cages. The failure load of the NiTi-l cage slightly exceeded that of the NiTi-s cage, although this difference was not statistically significant. Conform previous studies, a direct relation between force at failure and BMD was found in this study (Bisschop et al., 2012; Hussein et al., 2013).

The primary subsidence in the vertebra was the result of the implantation of the cage spikes into the bone, and proved to be approximately 1.2 mm. This result has been thought to be necessary for good adhesion of the device to the endplate and for optimal stability. Secondly, the primary subsidence might stimulate bone fusion because of the additional compression of the bone graft between the vertebrae. In order to prevent loss of height by subsidence of the two-sided spikes, an instrument can be used to enlarge the intervertebral space during the implantation of the MAC cage.

To prevent failure after intervertebral cage implantation in patients, knowledge about the minimum required load is warranted. The failure load of the MAC cages proved to be 2043 N for the NiTi-l cage, and 1866 N for the NiTi-s cage. The study of Wilke et al. (2001) measured intradiscal pressure in a non-degenerated L4–5 disc of a 45 year old male volunteer and discovered that intradiscal pressure depended on the kind of preceding activity, posture, external loads, and muscle activity (Wilke et al., 2001). Kandziora et al. (2002) reported that the L4-L5 motion segment of a 80 kg weighing patient could experience peak loads on the order of 2.24kN (Kandziora et al., 2002). Regarding the results of failure load from our study, this suggests that a risk of failure under physiological loads is present. However, considering the use of only small size devices in this study and the additional biomechanical strength obtained from the bone graft and posterior stabilization with pedicle screws, the MAC cages might therefore still meet the threshold for short-term as well as long-term clinical stability. Future studies should be performed to prove this statement.

In cylindrical cages generally higher failure loads have been observed after axial compression tests in cadaveric human spines (Kandziora et al., 2002). For example, the failure load observed with a threaded, hollow, porous titanium BAK cage with a diameter of 15 mm and a length of 24 mm averaged 7.42kN (Kandziora et al., 2002). However, several complications have been reported in the use of BAK cages, including risk for long-term subsidence and corrosive effects, that contribute to an increase in revision surgery (Beutler & Peppelman Jr., 2003). For this reason, interbody devices with large graft spaces for optimal bone fusion are warranted. Although vertical cages like the MAC cage have lower failure loads, they certainly provide larger graft spaces and might therefore prolong the survival of the PLIF construction. The MOSS vertebral body replacement spacer (“Harms mesh cage”) is another example of a vertical cage.

The study of Knop et al. (2001) showed a mean maximum compressive force of 2.72kN for this device, which exceeds a little the failure load of the MAC cage (Knop et al., 2001). However, the contact area of the MOSS was 302mm^2^, whereas the NiTi-l and NiTi-s only have a contact area of 30.4 mm^2^ and 15.04 mm^2^, respectively. The MAC cages thereby, and through their wide and open design, offer a larger space for additional bone grafts. It has been well known that the surface area between graft bed and bone graft is one of the most important factors for optimal spinal fusion and secondary stability. Interbody graft area should be significantly greater than 30% of the total endplate area to provide sufficient compressive strength (Closkey et al., 1993; Sukovich, 2004). Many cage designs do not provide such graft area, with the result that the contact area of local bone inside the cage might be insufficient for load transmission (Lee et al., 2010). Furthermore, the study of Lee et al. (2010) determined contact areas of fused local bone inside titanium cages using 3-dimensional thin-section computed tomography, and discovered that the ratio of fused area of local bone to total graft area inside cages was less than 50% (Lee et al., 2010). Although a fusion rate of 96.2% was reported, this indicates that only < 50% of the exposed graft area inside cages contributes to the compressive strength of the bone fusion. For these reasons, MAC cages could possibly provide an relevant advantage with their small cage contact area and large graft space in comparison with other vertical cages. However, this statement is for the time being based on an assumption and future research should determine which vertical cage design is superior for optimal spinal fusion. Considering the smaller cage contact area and thus larger graft space of the NiTi-s design, this cage type is favoured over the NiTi-l and should be used in further research study’s. The minimal invasive introduction of the MAC cage leaves the posterior longitudinal ligament largely intact and a large surrounded area for impacting the bone graft.

The acute subsidence at a force of 800 N and the failure load were comparable between the two MAC cages. Considering the small cage contact area, relatively high axial force was needed for displacement in the vertebral body. This could be explained by the close position of implant to the cortex of the vertebra. A limitation in the current study was the inaccuracy of the measurement of subsidence. A spondylodesis is often performed in a degenerated spine without equal surfaces of endplates. Therefore, the amount of subsidence probably differs over the surface area while in this study only the mean subsidence in the vertebral body was measured.

A second limitation of the current study was that only an axial compression test was performed in cadaveric models. The flexion, extension, bending, and rotational stiffness or range of motion of the MAC cage designs were not evaluated. However, it has been thought that segmental stability will largely depend on the postior fixation with pedicle screws and rods system. This was confirmed by the study of Wang et al. (2014). They showed a statistically significant improvement in stability when the cage or bone graft was supplemented with posterior instrumentation compared to biomechanical tests with cages alone (Wang et al., 2014). With this and the spikes on the edges and the wedge shape of the MAC cage design in consideration, we did not expect clinical relevant limitations in stability of the construction, and therefore only performed axial compression tests.

Finally, a limitation could be the fact that these tests only reflect short-term subsidence. The long-term biomechanical strength of the design is unknown and difficult to test in vitro. Due to the small cage contact area of the MAC cages in comparison with other cages, it is highly likely that this design will fail earlier without additional bone graft supplementation. Therefore, good bone ingrowth is a prerequisite for a successful PLIF construction.

## Conclusion

In conclusion, the biomechanical strength of both NiTi-l and NiTi-s cages is good and comparable to each other with a limited amount of short-term subsidence after the initial implantation of the cage spikes into the bone.

## Abbreviations

BMD: Bone mineral density
MAC: Memory Metal Minimal Access Cage
PLIF: Posterior lumbar interbody fusion
